# A Conversation with Paul Anastas

**DOI:** 10.1021/acscentsci.2c01055

**Published:** 2022-09-13

**Authors:** Craig A. Bettenhausen

Paul Anastas is matter of fact about the influence he and his collaborators have had on science. It was always their intention to change the world through green chemistry.

An organic chemist by training, Anastas maintains an active research program at Yale University that spans chemistry, chemical engineering, environmental sciences, epidemiology, and related disciplines. He also codirects the Yale Center for Green Chemistry and Green Engineering.

**Figure:**
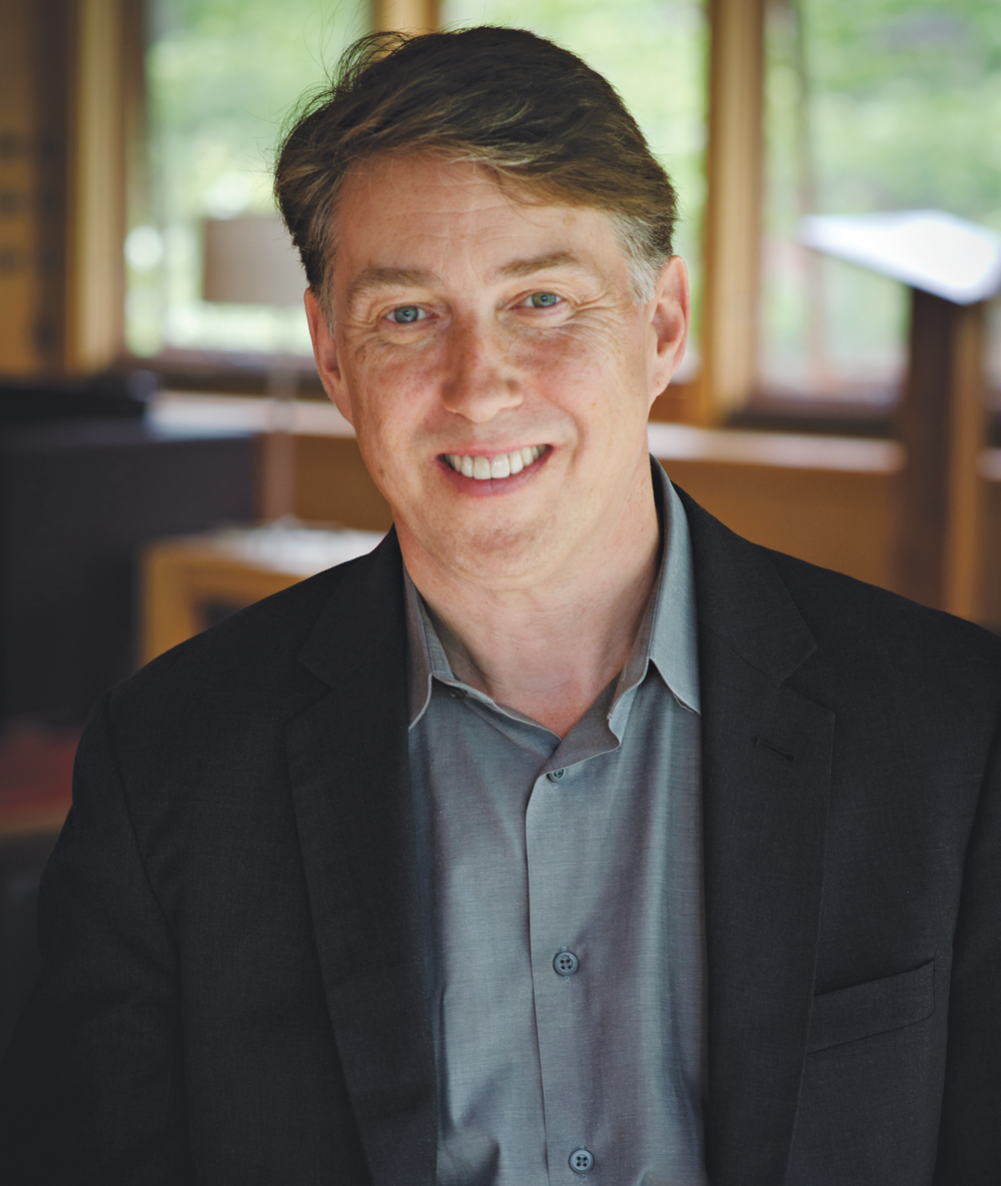
Credit: Yale School of Public Health

Academia is only part of his story. He spent almost 20 years in government service, culminating in a three-year appointment by former President Barack Obama to lead R&D at the U.S. Environmental Protection Agency. He’s active in the business of chemistry, including holding founder and board roles in start-ups such as the personal care ingredient maker P2 Science and the carbon dioxide-to-chemicals firm Air Company. He also has consulting contracts with Fortune 100 firms. As an advocate, he’s championed federal legislation encouraging the adoption of green chemistry principles in government-funded research.

On September 1, Anastas and frequent collaborator John Warner received the August Wilhelm von Hofmann Commemorative Medal from the German Chemical Society in recognition of the lasting impact of the pair’s 1998 book *Green Chemistry: Theory and Practice*, which laid out the 12 principles of green chemistry, and their subsequent work fleshing out those concepts. C&EN interviewed Anastas via video call in mid-July. This conversation was edited for length and clarity.

## The early publications on green chemistry had a heavy emphasis on synthetic design. What are some of the most interesting ways green chemistry has changed?

If, in 1991, I had come out and said, “You know, the way we’re doing things is all wrong. We have to completely rethink everything from feedstocks to life-cycle impacts to degradation to synthetic methods. And by the way, we have to think of it in terms of the entire system all at once,” no one would have embraced that. What we needed to do was show how it could be real to people. If somebody was going to increase their atom economy, fantastic! And somebody was going to move from a toxic solvent to a benign solvent or even use water, fantastic, right? So you celebrate those pieces. But over time, we always knew that this is a systems issue that cuts across the entire life cycle. This is about innovation and new ways of thinking about chemistry.

People soon figured out that it’s not about what you can’t do or what you need to stop doing. It’s what you can invent, can create: new materials, new synthetic methodologies. The definition of *green* in the dictionary is “young, fresh, new.” And there’s so many new platforms out there that have come out of green chemistry; that’s perhaps the biggest part of the evolution.

## Many of your students and collaborators can be found leading companies, research labs, and generally expanding the meaning and remit of green chemistry. What’s it like for you as a mentor and teacher to see people build on your ideas, take them in new and different directions?

That’s the whole point! If you go back through the literature, you’ll see that there’s a lot of one-off papers, and quite frankly, that’s intentional. So if you look back into the ’90s, more than 20 years ago, you’ll see papers about catalysis as a pillar of green chemistry, papers on green analytical chemistry, green chemistry in solids; these things are meant to flag different areas of green chemistry.

We often sell technologies. But it’s more important to spin out the chief technical officer. I’ve been so lucky to have such amazing entrepreneurial students like Patrick Foley, a founder of P2 Science; Stafford Sheehan, a founder of Air Company; and Laurene Petitjean, a founding chemist at the cooling-tower direct-air carbon-capture firm Noya. And I look at so many others who are doing the same thing. So there is this wonderful collection of innovation. It’s hard for me to think of a sector that doesn’t get touched by green chemistry.

## You recently took on a formal board role at Air Company, which is making ethanol from carbon dioxide. How does a new chemical route to vodka embody green chemistry?

I look forward to a day 50 years from now, when I look back on all these great inventions in the early part of the 21st century, and perhaps the greatest of all will be that people figured out how to take our biggest problem—our civilization-wide problem of CO_2_ waste changing our planet—and turn it into one of our greatest solutions. And that is using CO_2_ as a building block for the material basis of our society and our economy.

When Air Company came out with a luxury vodka, people would say to me, “Hey, all of the vodka in the world isn’t going to cure climate change!” But that’s not the point. If you can capture people’s imaginations, they can understand that you can turn CO_2_—one of our most impactful pollutants—into a luxury vodka. Then it becomes easy for them to understand that you can turn it into other things at quite large scales, like building materials and fuels. The point was to capture people’s imaginations, and that’s what’s happening.

## The use of microbes and enzymes instead of conventional chemical synthesis is a hot area in research and business right now. How can folks incorporate green chemistry into bioprocessing?

We’re all talking about molecular transformation. Enzymatic transformations have been part of green chemistry since the very beginning. The advantages include that you have, in some cases, ultraselectivity; you’re doing it in a benign solvent; you’re often using, by necessity, renewable feedstocks. The same rules and framework apply. But you also have to seriously consider things like energy, the overall atom economy and step economy, and what happens to waste at the end.

My codirector of the center here is Julie Zimmerman. Her work on the integrated biorefinery is learning the lessons from the petroleum refinery. The way petroleum conquered the world was not by saying, “We have a black goo, and we’re going to turn it into gasoline.” It was by saying, “We’ve got a starting material, and we are going to get value out of every fraction of that feedstock.” Biofuels and biomaterials used to be about producing a single product, whereas today there is an emerging approach where it starts with biomass and squeezes value out of every fraction of that feedstock. With almost any plant-based feedstock, you’re able to get lipids, you’re going to get some proteins, and you’re going to get very small-volume, high-value substances. All those things contribute to making the overall economic case for the biorefinery, the same way it did for the petroleum refinery 100 years ago.

## Practically every chemical company talks a big sustainability game these days. But how do you tell who’s really doing the work and who’s mostly talking from the marketing budget?

There is good news: just about every company now is doing some kind of green chemistry. They’ll have a particular product line or manufacturing process or something like that. I always said the whole term “green chemistry” will disappear when it’s just the way we do things. We’re not quite there yet. But we’re moving there even faster than I expected. Not as fast as I had hoped, but faster than I expected.

So how do you tell? It doesn’t take more than a few questions. Are they looking at the entire life cycle? If they say, “No, we’re concerned about what happens in our factory.” Well, that’s nice, but that’s not where you need to be. You ask if their measures and incentives are aligned with green chemistry. It’s nice to put up goals, but if you don’t actually incentivize it and measure, then so what? And you ask if they actually have any green chemists on staff. That’s a clear sign—if they’re working with green chemists, that makes all the difference.

There are companies that aren’t talking about it because it’s giving them a competitive advantage. As much as I want every company to talk about what they’re doing and share best practices and things like that, you don’t just share the crown jewels. The fact that green chemistry is giving companies a competitive advantage is a good thing. It might happen a little bit slower, but it’s going to stick.

## Craig Bettenhausen is an associate editor at

*Chemical & Engineering News*, *the independent news outlet of the American Chemical Society. A version of this story appeared in C&EN.*

